# Alternative Erythropoietin Receptors in the Nervous System

**DOI:** 10.3390/jcm7020024

**Published:** 2018-02-02

**Authors:** Daniela Ostrowski, Ralf Heinrich

**Affiliations:** 1 Department of Biology, Truman State University, Kirksville, MO 63501, USA; 2 Department of Cellular Neurobiology, Institute for Zoology, Georg-August-University Göttingen, 37073 Göttingen, Germany

**Keywords:** erythropoietin, non-hematopoietic functions, neuroprotection, regeneration, alternative erythropoietin receptors, common β chain receptor, ephrin B4 receptor, cytokine receptor-like factor 3

## Abstract

In addition to its regulatory function in the formation of red blood cells (erythropoiesis) in vertebrates, Erythropoietin (Epo) contributes to beneficial functions in a variety of non-hematopoietic tissues including the nervous system. Epo protects cells from apoptosis, reduces inflammatory responses and supports re-establishment of compromised functions by stimulating proliferation, migration and differentiation to compensate for lost or injured cells. Similar neuroprotective and regenerative functions of Epo have been described in the nervous systems of both vertebrates and invertebrates, indicating that tissue-protective Epo-like signaling has evolved prior to its erythropoietic function in the vertebrate lineage. Epo mediates its erythropoietic function through a homodimeric Epo receptor (EpoR) that is also widely expressed in the nervous system. However, identification of neuroprotective but non-erythropoietic Epo splice variants and Epo derivatives indicated the existence of other types of Epo receptors. In this review, we summarize evidence for potential Epo receptors that might mediate Epo’s tissue-protective function in non-hematopoietic tissue, with focus on the nervous system. In particular, besides EpoR, we discuss three other potential neuroprotective Epo receptors: (1) a heteroreceptor consisting of EpoR and common beta receptor (βcR), (2) the Ephrin (Eph) B4 receptor and (3) the human orphan cytokine receptor-like factor 3 (CRLF3).

## 1. Introduction

The helical cytokine erythropoietin (Epo) is an evolutionary ancient protein that is present in all major vertebrate lineages and can be synthesized by many cell types [1,2]. The name “erythropoietin” (first mentioned by Bonsdorff and Jalavisto [3]) arose from its functional implication in the generation of red blood cells (erythrocytes) to improve tissue oxygen supply, which was first described with some comprehensiveness by Erslev [4]. Cytokines typically mediate diverse responses in different tissues that may vary depending on their concentration, duration of exposure, developmental status and physiological context and may involve different types of receptors [5,6]. Beyond its role in erythropoiesis, numerous production sites, pleiotropic functions and diverse stimuli that induce Epo production were identified in mammalian and other vertebrate tissues, providing a basis for the hypothesis that Epo signaling originally evolved as a general mechanism to maintain or re-establish cellular functions under challenging physiological conditions, following injury and during pathogen invasion [7,8,9,10,11].

In addition to fetal liver and adult kidney, which in humans account for most of the circulating hormone, Epo is locally produced and released by cells of various tissues including heart, spleen, lung, testis, ovaries, retina and the nervous system (reviewed by: [12,13,14,15]). Within the nervous system, astrocytes, oligodendrocytes, neurons and endothelial cells may release Epo as a paracrine and/or autocrine signal (reviewed by [16]). Circulating, hormonal Epo is a four-helix glycoprotein consisting of 165 amino acids and several chains of carbohydrate residues that make up ~40% of the mass of approximately 34 kDa. Mammalian brain-derived Epo has a lower content of sialic acid residues and brain-specific Epo splice variants have been detected in fish [17]. In addition to full length Epo, splice variants of human and murine Epo have been detected, among them the exon 3 deletion variant EV-3 present in human serum that lacks erythropoietic activity but protects various types of neurons from apoptotic cell death [18,19,20]. Similar to the production of circulating Epo (reviewed by [21]), hypoxia also increases Epo production and release in the nervous system, but various other challenges or insults have also been demonstrated to induce Epo in the nervous system, such as mechanical damage [22,23], infection [24], metabolic stress [25], elevated temperature [26], intense neural activity [27,28] and enriched environment [29]. Prominent general functions of Epo in the nervous system and in other non-hematopoietic tissues are protection from apoptosis, reduction of inflammatory responses and re-establishment of compromised functions by support of proliferation, migration and differentiation to compensate for lost or injured cells [12]. Concerning the nervous system more specifically, Epo has been demonstrated to be crucial for normal brain development [30,31], to act neuroprotectively after hypoxic/ischemic insults and glutamate excitotoxicity [28,32], to suppress neuroinflammatory processes including activation of microglia [33,34] and to promote regeneration after axonal damage [35,36,37]. Moreover, Epo enhanced cognitive performance and memory retrieval in healthy humans and patients affected by mood disorders or schizophrenia [38,39,40,41,42,43] paralleling experimental observations of wild type rodents, rodent models for neuropsychiatric diseases and diabetic mice [44,45,46,47]. Part of these effects on cognitive performance may result from Epo-induced increase of hippocampal pyramidal neurons and oligodendrocytes, which is only maintained when respective brain regions are sustainably challenged [48], and from Epo-mediated elevation of general motivation [49,50].

It is meanwhile widely accepted that Epo mediates various beneficial effects on the development, maintenance and regeneration of nervous systems and options to use Epo or Epo derivatives for the treatment of neuropsychiatric and neurodegenerative diseases are being explored. In contrast, identification of the molecular nature of the involved Epo receptors has not been completed. A great number of (partially inconsistent) experimental results support the existence of a “tissue-protective” Epo receptor that differs from the homodimeric EpoR on erythroid progenitor cells. Since this proposed “tissue-protective” Epo receptor is not universally expressed in all cells that exhibit beneficial responses to Epo stimulation, other Epo-binding receptors must exist and expression of particular types of Epo receptors might generally depend on cell type, developmental stage and physiological context. This review summarizes evidence for the involvement of homodimeric EpoR and alternative Epo receptors as initiators of protective and regenerative mechanisms in non-hematopoietic tissues, with focus on the nervous system.

## 2. Epo Function in Non-Hematopoietic Tissue Other Than the Nervous Tissue

Besides the nervous system, Epo mediates a variety of functions in other non-hematopoietic tissues (reviewed by [16,51,52]). In the kidney interstitial fibroblasts produce and release hormonal Epo into the blood stream. However, human and rodent kidney also express EpoR, indicating an additional paracrine function of Epo [53]. Epo mediates a direct cell protective function following ischemia-reperfusion injury in rat kidneys [54,55] and improves kidney function in a rat model for chronic kidney disease [56]. Epo also has a direct proliferative effect on mouse (not rat or human) myoblasts from skeletal muscle fibers and protects myoblasts against apoptosis in vitro [57]. In vivo studies using rodent models indicate that Epo-EpoR signaling can facilitate skeletal muscle repair following cardiotoxin-induced and mechanically induced muscle injuries [58,59]. In humans, improved motor function in Friedreich Ataxia patients following prolonged Epo administration has been reported [60]. Similar protective effects of Epo have been shown in animal models for ischemia-reperfusion injury of the heart [61,62] and for chronic heart failure [63], indicating a cardioprotective activity of Epo. The cardioprotective effect of Epo is mediated by a direct anti-apoptotic effect on cardiomyocytes as well as enhanced Epo-induced neovascularization due to the mobilization of endothelial progenitor cells from the bone marrow [63,64,65,66].

The angiogenic potential of Epo is also implied in other physiological processes and pathophysiological conditions. Epo is produced in cells of the uterus in an estrogen-dependent manner to promote blood vessel formation [67]. Epo promotes skin wound repair, especially in diabetic rodents and humans [68,69]. Besides angiogenesis, Epo exerts proliferative effect on numerous skin cell types, stimulates coagulation, reduces the inflammatory response, facilitates blood vessel regeneration, and enhances endothelial cell mitosis (reviewed by [70]). The role of Epo in tumor growth has been discussed as well, since many tumors express EpoR. Epo is widely used to treat anemia in patients undergoing chemotherapy [71] but its anti-apoptotic, proliferative and angiogenic effect might have a growth-promoting effect on cancer cells (reviewed by [72]). More recently the role of Epo-EpoR signaling in the regulation of energy metabolism has been described. Adipose tissue expresses EpoR and mice with selective EpoR knock out within adipose tissue show reduced total activity and develop obesity and insulin resistance [2,15,73]. The described functions of Epo in non-hematopoietic tissues could be mediated by both circulating Epo, predominantly produced by the kidney or other endocrinal organs, and tissue-derived, locally produced Epo.

## 3. Homodimeric EpoR

### 3.1. Presence and Function of EpoR in the Mammalian Hematopoietic System

Epo-EpoR signaling is the key regulator for the formation of mature erythrocytes (erythropoiesis) [21,74]. The adequate production of erythrocytes is important during all stages of life. Primitive erythrocytes are formed in yolk sac blood islands and express EpoR, from embryonic day 7.5 in mice and ~16–20 days of gestation in humans [75,76]. Although Epo is not crucial for the progress of differentiation of primitive erythroblasts, Epo supports their proliferation and survival [75,76].

Later in development erythrocytes are continuously formed from pluripotent stem cells located in the fetal liver and adult human bone marrow and mice spleen (definitive erythropoiesis; reviewed by [77]). The maturation and differentiation of erythrocytes involves a stepwise differentiation process of hematopoietic progenitors that is controlled by the interplay of various cytokines, including Epo and e.g., stem cell factor (SCF) [78]. EpoR is expressed at low levels on burst-forming unit-erythroids (BFU-E) and its expression levels increases up to 10× on colony-forming unit-erythroids (CFU-E). Elevated expression of EpoR is mediated by transcription factors, such as GATA1, SCL/TAL1 and EKLF and Epo itself [75,79]. Epo and EpoR knock-out mice showed a complete impairment of erythropoiesis beyond the formation of CFU-E cells, indicating that Epo-EpoR signaling is crucial for cell survival and differentiation during this stage of erythropoiesis [80]. Later in erythropoiesis EpoR is downregulated and not required for cell survival.

### 3.2. Molecular Characterization of EpoR

EpoR expressed on the surface of immature erythroid cells is a member of the type I cytokine receptor family. In absence of the ligand, two receptor monomers form a disulfide-linked dimer that is integrated in the cell membrane. EpoR is comprised of an extracellular region, a single transmembrane domain, and an intracellular domain. The extracellular region consists of two domains (membrane-distal D1 and membrane-proximal D2) that provide two discrete binding sites for Epo. Initial binding of Epo to the high affinity site is followed by binding to the lower affinity site on the second monomer, which results in a conformational change of the dimeric EpoR [81,82]. Binding of Epo to both sites seems to be equally important for signal transduction, since mutation in one of the sites is sufficient to impair Epo-mediated signaling [82,83]. The first step of signal transduction following the conformational change of the receptor involves phosphorylation of several tyrosine residues within the membrane-distal portion of the intracellular domain. Since the receptor itself lacks kinase function, phosphorylation is mediated by a protein tyrosinase kinase (Jak2) that is constitutively associated with the receptor [84,85]. The main signal transducers for EpoR are signal transducer and activator of transcription factors (STAT) 5A and 5B that are activated within seconds after Epo binding [86,87]. STAT5A and STAT5B then accumulate in the nucleus and mediate Epo-responsive transcription of genes that control processes such as proliferation, apoptosis and cell differentiation. Other signaling pathways initiated by EpoR have also been reported, including the phosphoinositide-3-kinase (PI3K-AKT) and mitogen-activated protein kinase (Ras/MAPK) [88,89,90,91,92]. Negative feedback modulators from the suppressor of cytokine signaling family (SOCS) limit the function of EpoR signaling by acting on Jak2 and preventing STAT activation [93,94].

Key aspects of the hematopoietic EpoR structure and Epo-EpoR signaling are well conserved within vertebrates. The overall comparison of the zebrafish EpoR sequence to other vertebrates is relatively low (ranges from 44% to 27%), however essential sites that are required for receptor dimerization, Epo binding, conformational changes and intracellular docking sites for downstream signaling molecules are conserved [95]. Cytokine-Jak-STAT signaling is an evolutionary ancient mechanism. The fruit fly *Drosophila melanogaster* contains a typical cytokine receptor (*domeless*) as well as Jak and STAT homologues (*hopscotch*, *stat94/marelle*) that share functional similarities to vertebrate type I cytokine receptor signaling [96,97]. However, orthologs of *EpoR* have not been identified in *Drosophila* or any other invertebrate species and Epo-mediated neuroprotection (which is absent in *Drosophila* but present in other insects (see below)) has to rely on other types of Epo-responsive receptors [98,99,100].

### 3.3. Presence and Function of EpoR in the Nervous System

Observations of increased apoptosis in non-hematopoietic tissue in Epo or EpoR knock-out mice prior to the onset of anemia implied a functional role of Epo-EpoR signaling beyond erythropoiesis [101,102]. EpoR is expressed in a temporal and cell-specific manner in the developing brain [103,104], heart [105,106], kidney [53,107], skeletal muscle [108] and endothelial cells [109,110]. In the mouse brain, EpoR is expressed in the neural tube (in radial glial cells) as early as E8 at levels comparable to adult hematopoietic tissue [104,111]. In the developing human embryo EpoR expression is first detected, as early as 7–8 weeks, in neurons and astrocytes of the spinal cord and brain [103,112,113]. EpoR knock-out mice show a reduced overall amount of neuronal progenitor cells and reduced neurogenesis [114] as well as developmental defects of the heart accompanied with a reduction in the number of cardiac myocytes and endothelial cells [101]. Based on these results, Epo-EpoR signaling was proposed to play a crucial role for the normal development of the brain and other organs. Since knock-out of *Epo* and *EpoR* genes result in severe anemia and premature death at E13.5, the role of Epo-EpoR signaling in non-hematopoietic tissue was difficult to study until Suzuki et al. [115] generated a transgene-rescue EpoR knock-out mouse that rescued EpoR expression exclusively in the hematopoietic lineage (hematopoietic-rescued EpoR knock-out). Hematopoietic-rescued EpoR knock-out mice displayed increased apoptosis but revealed no apparent abnormalities in the gross structure of the brain [102,115]. However, in a more detailed analysis Chen and colleagues [116] described a 2-fold increased apoptotic rate in the brains of hematopoietic-rescued adult EpoR knock-out mice and cultured hippocampal neurons showed poor survival in contrast to comparable cultures from wild type hippocampus. In addition, induced myocardial ischemia-reperfusion injury in the same transgenic mice resulted in a significantly larger infarct size and increased apoptosis in cardiomyocytes compared to wild-type mice [61]. The hypothesized anti-apoptotic function of Epo-EpoR signaling in non-hematopoietic tissue was supported by various in vitro studies. For example, undifferentiated human neuroblastoma cells express EpoR and Epo treatment prevents augmented apoptosis following the exposure to pro-apoptotic stimuli. Upon differentiation into neuron-like cells (induced by treatment of neuroblastoma cells with all-trans-retinoic acid) EpoR was downregulated and Epo no longer had an anti-apoptotic effect [117,118]. Besides the expression of EpoR in neurons, its mRNA is also detected in cultured oligodendrocytes and astrocytes, and administration of Epo enhances oligodendrocyte maturation and astrocyte proliferation [119]. Furthermore, direct dose-dependent effects of Epo on the proliferation and survival of other non-hematopoietic cells, including cultured primary mouse myoblasts from skeletal muscle [57] and human proximal tubular cells from the kidney [107] has been reported.

In adults, neurogenesis is limited to regions of the hippocampus and subventricular zone (SVZ). Impaired Epo-EpoR signaling significantly reduces adult neurogenesis [116] and Epo administration to normal C57BL/6 mice resulted in an increased number of neurons and oligodendrocytes [48]. Interestingly, newly formed cells resulted from enhanced differentiation of pre-existing precursor cells rather than from the formation of new cells due to cell proliferation. In addition, overexpression of constitutively active EpoR in pyramidal neurons of forebrain cortex and hippocampus can increase synaptic plasticity and enhances cognitive abilities and social memory [120].

EpoR expression in adult nervous systems remains very low under normal/healthy conditions, but various factors can modulate EpoR levels. For example, in healthy rodents, environmental enrichment, ambient heat or mild episodes of hypoxia can increase EpoR expression and furthermore protect neurons towards following injuries including severe ischemia [25,26,29,121]. Similar to the brain, EpoR expression in adult kidneys and hearts remains low, but increases following ischemic insult [106,107]. Although hypoxia-inducible factor (HIF) does not directly induce EpoR expression, factors such as pro-inflammatory cytokines and Epo itself have been identified to modulate EpoR expression in non-hematopoietic tissues. Induction of EpoR expression in non-hematopoietic tissues following injury has been correlated with the tissue protective effect of Epo administration in a variety of disease models, including stroke [122,123], traumatic brain injury [23,124], hypoxic ischemic encephalopathy (HIE) in neonates [125,126,127], peripheral nerve injury [128,129], ischemic reperfusion injury of the heart [106,130] and kidney [54,55] and chronic kidney disease [56]. However, promising results from animal model studies have only been partially transferred into successful clinical human trials [131,132,133,134,135,136,137,138,139]. Severity of the injury, different dosages and time points of Epo administration might influence the outcomes of the clinical trials. In addition, the protective effect of Epo can only manifest if there is an appropriate receptor expressed on the cell surface. Though neuroprotective and neuroregenerative functions have been described to depend on EpoR, most studies did not address the question whether homodimeric EpoR or a heteromeric receptor complex that includes EpoR as one component relayed the Epo signal in the responsive cells. Future studies with specific Epo-mimetic ligands will need to analyze the role of EpoR in Epo-mediated neuroprotection in more detail.

It should be noted that methodical shortcomings in the association of EpoR expression with beneficial functions in the nervous system have led to partially inconsistent results about the role of EpoR in nervous tissue. The methods that have been used to detect EpoR in non-hematopoietic tissue include the use of EpoR antibodies in tissue sections and western blots; whole mount in situ hybridization and quantitative analysis of EpoR mRNA levels in tissue samples and tissue specific analysis of EpoR transcripts in transgenic mice that express the human EpoR transcript. Commercially available EpoR antibodies showed non-specific cross-reactivity and results of studies solely based on the use of these antibodies were questioned [140,141,142]. A recent study by Ott et al. [143] characterized a new, highly specific, EpoR antibody directed against the cytoplasmic tail of human and murine EpoR. Results of the study show a specific EpoR expression in cultured primary murine brain cells, frozen brain sections of healthy young mice, and upregulation of EpoR in injured mice brains and patients suffering from epilepsy. Future studies using more specific antibodies against EpoR will need to validate EpoR expression within other non-hematopoietic tissues. However, the overall evidence that EpoR mRNA is expressed by cells other than the hematopoietic system, the protective effect of Epo following tissue injury and the detection of specific binding sites for Epo in the brain support the hypothesis that Epo-EpoR signaling is functional in non-hematopoietic tissues.

### 3.4. Functions of Alternative Versions of EpoR

EpoR exists in three major isoforms that are generated by alternative splicing: full length protein as part of the functional receptor; a soluble protein that lacks the transmembrane and intracellular domains; and a truncated protein that lacks large parts of intracellular domains due to alternative splicing [144]. Some cells simultaneously express different isoforms of EpoR. The physiological role of both soluble and truncated EpoR has not been fully understood but both variants seem to be of functional importance.

Soluble EpoR (multiple variants may exist [8]) has been detected in various tissues including the brain [145]. Its presence in the blood is elevated by pro-inflammatory molecules [146] while its expression in the brain is downregulated (in contrast to full length EpoR) upon exposure to hypoxia [145]. Endothelial cells have been identified as one source of soluble EpoR within the mammalian brain [147]. Studies on the respiratory control system of mice indicated that the level of soluble EpoR, which captures Epo and prevents it from activating membrane-bound EpoR, regulates the sensitivity of Epo/EpoR signaling in the adaptation of ventilatory functions to different levels of oxygen [145]. Clearing of Epo from the circulation and extracellular space and regulating the availability of Epo for binding to membrane-bound Epo receptors seem to be the general function of soluble EpoR.

Truncated variants of EpoR have been identified in early-stage erythroid progenitor cells where they function as negative regulators of erythropoiesis [148,149]. Evidence for the function of truncated EpoR in the brain resulted from studies on substantia nigra dopaminergic neurons that co-express both, a full length EpoR and a truncated isoform that lacks large parts of EpoR’s intracellular domains [150]. While expression of full length EpoR in HEK293T cells enabled Epo-stimulated phosphorylation of STAT5, expression of the truncated isoform did not confer any Epo-sensitivity, suggesting that truncated EpoR may not form Epo-responsive receptor molecules. Coexpression of the full length EpoR and the truncated EpoR isoform prevented Epo-stimulated STAT5 activation, indicating interference of the truncated receptor with EpoR-initiated transduction [150]. While the factors that regulate the levels of full length and truncated EpoR expression in dopaminergic neurons remain to be identified, the level of truncated EpoR expression seems to regulate the sensitivity of these cells to Epo.

## 4. Alternative Epo-Receptors

While Epo prevents apoptosis of erythroid progenitors by activating homodimeric EpoR, its protective functions in the nervous system and other non-hematopoietic tissues are also mediated by alternative receptors. Though Epo has a lower affinity to tissue-protective receptors than to homodimeric EpoR [7], brief access of the ligand is sufficient to induce neuroprotection [7,28,151] while stimulation of erythropoiesis via homodimeric EpoR requires prolonged exposure to the ligand. This difference may result from different mechanisms to inactivate the respective Epo receptors or components of their downstream transduction pathways (summarized in [152]). A study with EpoR conditional knock-down mice by Tsai and co-workers reported persisting Epo-mediated neuroprotection during ischemia [114]. Existence of multiple, sometimes tissue-specific Epo splice variants suggests different functions that might be mediated by different receptors in different tissues [19,153]. As one example, the human splice variant EV-3 which lacks the entire third exon of the full-length Epo transcript does not activate homodimeric EpoR, and hence does not stimulate erythropoiesis, but mediates neuroprotection of rat hippocampal and insect neurons [19,20]. In addition to natural splice variants, various Epo-derivatives (e.g., carbamylated Epo, Epobis, helix B surface peptide) and molecules completely unrelated to the Epo peptide sequence (e.g., STS-E412) have been identified as non-erythropoietic but neuroprotective specific Epo mimetics [154,155,156,157,158]. Recent studies identified three candidate receptors that are expressed in the nervous system and mediate cell protective and regenerative functions upon binding of Epo and/or one of its non-hematopoietic agonists.

## 5. Common β Chain Receptor

Several studies (examples see below) indicated that a heteromeric complex consisting of one or more EpoR together with one or more molecules of the common beta receptor chain (βcR) may serve as a tissue-protective Epo receptor.

βcR (synonym CD131) is a member of the type I cytokine receptor subfamily, which forms heteromeric receptors with high-affinity receptor subunits for interleukin-3, interleukin-5 and granulocyte-macrophage colony-stimulating factor (GM-CSF) involved in particular regulatory mechanisms of hematopoiesis [151,159,160]. In these receptor complexes that include one “specific” and two βcR subunits, βcR is essential for receptor signaling, which is initiated by ligand-induced phosphorylation of its intracellular domains. Similarly (although stoichiometry may be different), βcR also associates with EpoR to form an Epo-sensitive tissue-protective heteroreceptor [6,7,154,161,162], which may already assemble in the absence of Epo ligand [163]. Co-expression of EpoR and βcR has been detected in various cell types of the nervous system, the heart, the kidney and other organs (summarized in [144,157]) and Epo has been reported to induce tyrosine phosphorylation of βcR [164]. Since both homodimeric EpoR and EpoR/βcR complexes activate Jak, downstream intracellular signaling pathways of both receptors are typically similar, though differences have been reported in a study on mouse primary neurons [165]. Like EpoR, large portions of βcR are typically localized in intracellular compartments and their exposure at the cell surface is stimulated by physiological stressors such as hypoxia, metabolic deficiencies and inflammation [165,166]. Mechanisms that could regulate a preferential formation of homodimeric EpoR/EpoR versus formation of EpoR/βcR complexes from co-expressed subunits have not been characterized.

In the nervous system, cell types that can co-express EpoR and βcR include neurons, astrocytes, microglia and vascular cells. Epo-mediated neuroprotection through EpoR/βcR has been demonstrated in various studies [158,162,167,168] including studies with βcR-deficient knock-out mice that lacked Epo-mediated neuroprotection [162] and relief of neuropathic pain [169]. In addition, Epo mediates cardio- and reno-protective effects through EpoR/βcR-signaling in vitro and in vivo [162,170]. Selective activation of heteromeric EpoR/βcR but not of homodimeric EpoR has been demonstrated for CEpo [154], peptides derived from or related to helix-B of Epo [151,171] and several other ligands including mutant versions of Epo. Cells that co-express EpoR and βcR should contain both heteromeric EpoR/βcR and homodimeric EpoR receptors. While Epo and other unselective ligands may induce activation of various transduction pathways starting from both types of receptors, ligands selective for heteromeric EpoR/βcR complexes may only evoke some portion of the response spectrum induced by Epo [167]. However, various brain regions and cell types that exhibit Epo-mediated protection do not co-express EpoR and βcR in detectable amounts [29,172,173] and other neuroprotective but non-erythropoietic signals (such as the Epo splice variant EV-3) have been demonstrated to bind neither homodimeric EpoR nor EpoR/βcR receptors [19]. This indicates that heteromeric EpoR/βcR (in addition to homodimeric EpoR) functions as a protective Epo receptor, at least in some types of neurons and glia and eventually depending on the type of physiological challenge, but certainly additional protective receptors exist in the mammalian nervous system.

## 6. Ephrin B4 Receptor 

Ephrin receptors (Eph) represent the largest subfamily of receptor tyrosine kinases that typically mediate contact dependent cell-to-cell communication through interactions with membrane-bound ephrin ligands (reviewed by: [174,175]). Like Epo/EpoR signaling, ephrin/Eph signaling is involved in hematopoiesis, vascularization, cancer cell regulation and various functions in developing and mature nervous systems [176]. In the mammalian nervous system ephrins and Eph are expressed by neurons, astrocytes and endothelial cells [177,178,179]. Ephrin/Eph signaling regulates neurogenesis, axon growth, cell migration, synapse formation, dendritic spine morphology and synaptic plasticity underlying long-term changes of synaptic strength and memory formation [177,180,181,182,183]. Mammalian ephrins and Eph are divided in two classes A (6 A-ephrins and 9 EphA) and B (3 B-ephrins and 5 EphB) and within a class Eph typically can bind different ephrins (“promiscuous binding”). The stoichiometry of ephrin/Eph complexes seems to be variable. Preformed ephrin dimers and Eph dimers initially form heterotetramers which may associate to multimeric complexes whose particular composition can initiate specific responses in either one or both interacting cells (reviewed by [184]).

The EphB4 receptor differs from other Eph receptors by containing an isoleucine instead of a tyrosine at position 48 in the hydrophobic cavity. Since EphB4 has been demonstrated to interact rather specifically with ephrin-B2 [184] it might appear surprising, that just EphB4 also serves as a functional receptor for Epo [185]. Studies on ovarian carcinoma cells, that endogenously express both EphB4 and EpoR, demonstrated that both ephrin-B2 and Epo directly activate EphB4, causing increased proliferation and invasive migration mediated by Scr kinase (a cytosolic non-receptor tyrosine kinase) and STAT3 [185]. In contrast, EpoR activation in the same ovarian carcinoma cells leads to Jak activation and elevation of STAT5. Direct activation of EphB4 by Epo was further confirmed in EphB4-transfected COS-1 cells that did not express EpoR endogenously. Consequently, both ephrin-B2 and Epo independently activate EphB4 and may act synergistically when released onto the same target cells. Binding studies characterized EphB4 as a low affinity receptor for Epo with a KD of 880 nM compared to a KD of 28 nM for EpoR expressed in the same cells [185]. Effective activation of EphB4 may therefore critically depend on the relative abundance of EphB4 and EpoR on the cell surface (which is generally very low for EpoR in non-erythroid cells) and the sufficiency of short ligand exposure to activate downstream transduction mechanisms. However, data on both the surface expression of EphB4 and the time course of EphB4-initiated processes are not available. Moreover, survival of patients with breast and ovarian cancers was reduced with increased expression level of EphB4 but not of EpoR, and Epo treatment further reduced the survival chances of breast cancer patients with tumors that express high levels of EphB4 [185]. This indicated that Epo supported tumor growth particularly by activation of EphB4-initiated mechanisms.

EphB4 is expressed in the mammalian nervous system and ephrin-B2/EphB4 signaling has been demonstrated to regulate adult neurogenesis and its balance with gliogenesis in the subgranular zone of the hippocampus [177,186]. EphB4 expressed by neural stem cells is activated by direct contact with ephrin-B2 expressing astrocytes. This interaction stimulates self-renewal of and proliferation of neural stem cells and differentiation towards the neuronal lineage [177,186]. Indicating its role in synaptic function, ephrin-B2 knock-out in mouse hippocampus attenuated long term potentiation of synaptic transmission (LTP) following high-frequency synaptic activation [187]. Since Epo signaling in the brain has also been associated with neurogenesis and/or neuronal differentiation [48,116,188] and increased cognitive performance [42,120,189] it may well be that part of these processes are mediated via activation of EphB4 receptors. In addition, rat cortex neurons were demonstrated to co-express EpoR and EphB4 (as in the carcinoma cells described above) which probably enables differential cellular responses initiated by the two receptors that can both be activated by Epo [185].

## 7. Cytokine Receptor-Like Factor 3 (CRLF3)

Human *CRLF3* (synonyms Crème9 and Cytor4; splice variants with minor differences) is an orphan cytokine receptor of unknown function that belongs to the subgroup of prototypic type 1 cytokine receptors which also includes EpoR, thrombopoietin receptor, prolactin receptor and growth hormone receptor [190]. The human *CRLF3* gene is located on chromosome 17 and the protein is expressed in various normal tissues including the nervous system with higher expression levels in embryonic compared to adult brains. Additionally, some tumor cell lines and freshly isolated tumor tissues contain elevated levels of CRLF3 [191,192] Human CRLF3 includes 442 amino acids and contains a number of characteristic predicted domains. These include the conserved cytokine receptor motiv (WSXWS), a single-pass transmembrane sequence, and a docking site for Jak (NCBI GenBank NP_057070.3) [100,190] being consistent with reported activation of intracellular transduction pathways involving STAT3 [192].

Orthologs of human CRLF3 exist in other mammals, non-mammalian vertebrates (amphibia and fish), cephalochordates, tunicates and some insects including the beetle *Tribolium castaneum*, the locust *Locusta migratoria* and the cricket *Gryllus bimaculatus* but not the fruit fly *Drosophila melanogaster* ([98,101], unpublished own data). While *EpoR* orthologs are generally absent in insects and other invertebrates, the presence of CRLF3 in different insect species coincides with the presence of Epo-mediated neuroprotection and protective effects are absent in *D. melanogaster* [10,98,100]. Direct proof that CRLF3 represents a functional neuroprotective receptor for Epo was achieved by studies with primary brain cell cultures from *T. castaneum* [100]. In these neuron cultures, serum deprivation and hypoxia induce apoptotic cell death that can be completely prevented by both Epo and its non-erythropoietic splice variant EV-3. Knock-down of CRLF3 expression by double stranded RNA interference abolishes Epo’s protective effects, indicating that CRLF3 functions as a neuroprotective receptor for Epo-like ligands in beetles [100]. Since direct binding of Epo and EV-3 to CRLF3 has not yet been confirmed, neuroprotective effects could also be mediated by a heteromeric receptor that contains CRLF3 as a crucial component for signal transduction. Epo-mediated neuroprotection of primary brain neurons from *L. migratoria* depends on Jak/STAT signal transduction [99], suggesting that insect CRLF3 activates similar intracellular pathways as its mammalian orthologs. Whether CRLF3 functions as a neuroprotective receptor for Epo and its non-erythropoietic splice variants in mammals including humans is under current investigation.

## 8. Conclusions

Endogenous Epo in the nervous system serves to adapt cells and neuronal circuits to physiological and pathological challenges ranging from normal activity-dependent mild reduction of oxygen availability and energy metabolites, via excitotoxicity to severe damage resulting from stroke, injury and inflammation. Numerous studies in humans and animals have documented Epo-mediated beneficial effects in the nervous system that secure cell survival, maintain functionality and support regeneration in deleterious physiological conditions. Epo-mediated mechanisms represent promising targets for pharmacological intervention with consequences of injury, neurodegenerative diseases and neuropsychiatric conditions. Besides homodimeric EpoR, βcR/EpoR, EphB4 and CRLF3 (evidence summarized in Table 1), additional Epo-receptors might be expressed by particular cell types in particular physiological conditions. Hence, Epo and Epo-like ligands may activate different signal transduction cascades depending on their concentration (while low concentrations of Epo are beneficial, too high concentrations may be deleterious for the same cells) and on cell type, physiological condition and type of insult. Identification of Epo receptor molecules expressed by specific cells in particular situations may allow the development of Epo-mimetics that specifically interfere with one (or at least few) of the pleiotropic functions of Epo in- and outside the nervous system.

## Figures and Tables

**Table 1:** Protective functions of classical EpoR and alternative Epo-receptors.

Receptor	EpoR/EpoR	EpoR/βc-R	EphB4	CRLF3
	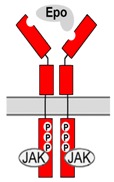	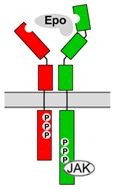	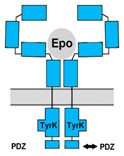	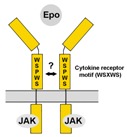
	Homodimer	Stoichiometry not clear or variable	Homodimer and multimeric complexes	Homodimer (and heteromers?)
Epo-mediated protection in/on	Erythroid progenitors Heart Skeletal muscle Kidney Brain Neuroblastoma cells various tumors PC12 cells transfected HEK cells	Kidney Heart Brain Macrophages Neuroblastoma cells transfected HEK cells Endothelial cells	Various tumors Carcinoma cell lines	Hemocytes (insect) *
Transduction pathways	Jak, STAT5, PI3K/AKT, Ras/MAPK, NF-κB (not in erythroid cells)	Jak, STAT5 PI3K/AKT MAPK	Scr tyrosine kinase, STAT3	Jak, STAT3, STAT (insect)
Alternative ligands:				
EV-3	No	No [19]	No data available	Yes [100]
carbamylated Epo	No	Yes [193]		No data available
helix b surface peptide	No	Yes [194]		Yes *
Expression in the nervous system	Neurons [28,116,143], Astrocytes [32,124,195], Oligodendrocytes [143], Microglia [143,195], Endothelial cells [32,110,143]	Neurons [158,162,166], Astrocytes [196], Endothelial cells [197]	Neural stem cells [177] Hippocampal neurons [187] Endothelial cells [179]	Neurons (insect) *
Epo-mediated effect within the nervous system	Neuroprotection of hippocampal neurons [172] and differentiated neuroblastoma cells [173]	Neuroprotection of rodent motor neurons [193] and spinal cord neurons [198]; Reduction of neuropathic pain in mice [169]	No data available	Neuroprotection of insect brain neurons [100]; Regeneration of insect auditory receptor fibers and neurites of cultured insect neurons *

Note: * own unpublished results. EpoR monomer of the classical erythropoietin receptor, βcR common beta chain receptor, EphB4 ephrin B4 receptor, CRLF3 cytokin receptor-like factor 3, Jak janus kinase, STAT signal transducer and activator of transcription, PI3K phosphoinositide 3-kinase, AKT protein kinase B, ras small GTPase protein, MAPK mitogen-activated protein kinase, NF-κB nuclear factor kappa B, Scr tyrosine kinase non-receptor protein kinase.
